# A Gly-β-muricholic acid and FGF15 combination therapy synergistically reduces “humanized” bile acid pool toxicity in cholestasis mice

**DOI:** 10.1016/j.jlr.2025.100936

**Published:** 2025-11-05

**Authors:** Mohammad Nazmul Hasan, Huaiwen Wang, Wenyi Luo, Yanhong Du, Lei Xiong, Lijie Gu, Tiangang Li

**Affiliations:** 1 Department of Biochemistry and Physiology, Harold Hamm Diabetes Center, University of Oklahoma Health Sciences, Oklahoma City, OK, USA; 2 Laboratory for Molecular Biology and Cytometry Research, University of Oklahoma Health Sciences, Oklahoma City, OK, USA; 3 Department of Pathology, Yale University, New Haven, CT, USA

**Keywords:** cholestasis, Cyp2c70, combination therapy, microbiome

## Abstract

Hydrophobic bile acid-mediated hepatobiliary injury is a major driver of cholestasis progression. Most anticholestasis treatments being tested clinically are based on a single agent, which does not always sufficiently alleviate bile acid toxicity to slow disease progression. This study investigates a therapeutic strategy of combining glycine-conjugated β-muricholic acid (Gly-βMCA) and fibroblast growth factor-15 (FGF15) to alleviate bile acid hepatobiliary toxicity in *Cyp2c70* KO mice that lack endogenous muricholic acid (MCA) synthesis and have a “humanized” hydrophobic bile acid pool composition. The effects of the single and combination treatments on bile acid metabolism, liver injury, and gut microbiome were investigated in female *Cyp2c70* KO mice with progressive cholangiopathy and portal fibrosis. While all three treatments significantly reduced biochemical and histologic features of liver injury, the Gly-βMCA and FGF15 combination achieved a remarkably higher reduction in both bile acid pool size and hydrophobicity than either single treatment. Mechanistically, this resulted from synergistically increased biliary hydrophilic MCA species derived from Gly-βMCA, inhibited intestine endogenous bile acid absorption by Gly-βMCA, and repressed cholesterol 7α-hydroxylase (CYP7A1) by FGF15, which counteracted the undesirable farnesoid X receptor antagonism activity of Gly-βMCA. Furthermore, a hydrophobic bile acid pool in *Cyp2c70* KO mice was associated with markedly reduced beneficial Lactobacillaceae family bacteria abundance, which was enriched by Gly-βMCA and the combination treatments. In conclusion, the Gly-βMCA and FGF15 combination shows enhanced efficacy in decreasing humanized bile acid pool size and hydrophobicity and holds potential as a therapeutic strategy to decrease bile acid burden in cholestasis.

Cholestasis is a pathological condition caused by genetic or acquired defects that impair bile flow out of the liver (1). The resulting accumulation of bile acids damages the liver parenchyma and bile ducts, which leads to increased risk of developing liver fibrosis, end-stage liver diseases, and liver and bile duct cancer. Alleviating bile acid toxicity is a major treatment goal for all forms of cholestasis. Currently, the hydrophilic bile acid ursodeoxycholic acid (UDCA; Ursodiol™) is the only available first-line drug for treating primary biliary cholangitis (PBC) (2). While UDCA can be a highly effective anticholestasis drug for PBC, up to 40% of the PBC patients did not adequately respond to UDCA, leaving them with limited therapeutic options (3, 4). The only second-line drug, obeticholic acid (Ocaliva), approved by the Food and Drug Administration in 2016, a potent farnesoid X receptor (FXR) agonist, has been shown to cause treatment-associated pruritus and hepatotoxicity (5, 6, 7, 8). Furthermore, there are no effective treatments for many other more severe forms of cholestasis, including primary sclerosing cholangitis and progressive familial intrahepatic cholestasis.

Bile acids are synthesized from cholesterol in the liver, and chenodeoxycholic acid (CDCA) and cholic acid (CA) are the two primary bile acids in humans (9). In the intestine, glycine-conjugated (Gly-) or taurine-conjugated (T-) CDCA and CA undergo deconjugation and subsequent 7-α-dehydroxylation mediated by bacterial enzymes to be converted to secondary bile acids, lithocholic acid (LCA) and deoxycholic acid (DCA), respectively. In mice, the murine-specific CYP2C70 enzyme converts most CDCA to α-muricholic acid (αMCA), which can be epimerized to β-muricholic acid (βMCA) in the liver (10, 11). Therefore, CA and MCA species are the major primary bile acids in mice. MCA species are 6-hydroxylated bile acids and therefore are highly hydrophilic and noncytotoxic, and their presence causes the mouse bile acid pool to be more hydrophilic and less toxic than the human bile acid pool. Genetic KO of the *Cyp2c70* gene in mice fully prevented MCA synthesis and caused the *Cyp2c70* KO mice to have a bile acid pool consisting of primary bile acids CDCA and CA, resembling the human bile acid pool composition (10). Interestingly, *Cyp2c70* KO mice developed spontaneous biliary injury and portal fibrosis, likely because of maladaptation to the “human-like” hydrophobic bile acid pool (10), which was more severe and progressive in female *Cyp2c70* KO mice and self-limited in male *Cyp2c70* KO mice (12, 13). In recent years, many research groups have used *Cyp2c70* KO mice as a human-relevant rodent cholestasis model for testing therapeutic approaches that alleviate bile acid hepatobiliary toxicity (12, 13, 14).

Currently, most of the anticholestasis treatments tested clinically are based on a single agent, which often exhibited insufficient therapeutic efficacy (7, 15, 16, 17, 18, 19, 20, 21). In contrast, the therapeutic efficacy of combining therapeutics with distinct mechanisms of actions is still an underexplored area of research. Intestine bile acid transporter apical sodium bile acid transporter (ASBT) inhibitors have been shown to reduce circulating bile acids and alleviate pruritus in cholestasis by reducing intestine bile acid absorption (22). Blocking intestine bile acid absorption induces compensatory bile acid synthesis in the liver. Another promising anticholestasis agent is fibroblast growth factor-19 (FGF19) analog, which inhibits cholesterol 7α-hydroxylase (CYP7A1), the rate-limiting enzyme in hepatic bile acid synthesis (15, 16). However, bile acid synthesis is often repressed in cholestasis because of intrahepatic bile acid accumulation, which may limit the additional benefit of pharmacological repression of bile acid synthesis. A previous study from our laboratory reported that simultaneous inhibition of hepatic bile acid synthesis and intestine bile acid absorption by a combination of ASBT inhibitor and fibroblast growth factor-15 (FGF15), the mouse ortholog of human FGF19, strongly reduced total bile acid pool and alleviated bile acid hepatotoxicity in female *Cyp2c70* KO mice (13). More recently, we have uncovered a potent anticholestasis effect of glycine-conjugated β-MCA (Gly-βMCA) (23, 24), a hydrophilic bile acid derivative that was identified by molecular modeling as a gut-restricted FXR antagonist with antiobesity and insulin-sensitizing properties (25). At the mechanistic level, we discovered that although Gly-βMCA was poorly absorbed in the intestine, it was extensively deconjugated in the large intestine and passively absorbed in the form of MCA. This unique bacteria-mediated pharmacokinetics resulted in an enrichment of T-MCA species in the endogenous bile acid pool, which lowered bile acid pool hydrophobicity and toxicity. We also discovered that the poorly bioavailable Gly-βMCA also inhibited intestinal absorption of endogenous bile acids and reduced total bile acid pool size, instead of expanding it. As an FXR antagonist, Gly-βMCA and its MCA derivatives induce hepatic CYP7A1 expression, which is an undesirable effect in cholestasis (14, 24). Based on the identified function of Gly-βMCA as a gut bile acid absorption inhibitor, we asked if Gly-βMCA and FGF15 could be used together as a combination therapy to simultaneously decrease bile acid synthesis, absorption, and hydrophobicity. This concept was tested in female *Cyp2c70* KO mice that developed severe and progressive hepatobiliary injury. Our findings demonstrated that the Gly-βMCA and adeno-associated virus (AAV)-FGF15 combination is a highly effective approach to simultaneously decrease bile acid pool size and hydrophobicity, which provides proof-of-concept evidence for a combination therapeutic strategy to treat human cholestasis.

## Materials and methods

### Reagent

Aspartate aminotransferase, alanine aminotransferase, and alkaline phosphatase assay kits were purchased from Pointe Scientific (Canton, MI). Gly-βMCA was purchased from MedChemExpress LLC (Monmouth Junction, NJ). The bile acid assay kit was purchased from Diazyme Laboratories (Poway, CA). Cytokeratin-19 (CK19) antibody (ab52625) was purchased from Abcam (Waltham, MA).

### Mice and treatments

The *Cyp2c70* KO mice on C57BL/6J genetic background with exon 4 deletion were generated as described previously (13). WT mice were obtained by breeding *Cyp2c70*^+/-^ mice. Female *Cyp2c70* KO mice developed progressive and more severe liver injury than male *Cyp2c70* KO mice (12, 13), which was the reason only female *Cyp2c70* KO mice were used to study the enhanced efficacy of the combination treatment. Mice were housed in microisolator cages under a 7 AM to 7 PM light cycle and a 7 PM to 7 AM dark cycle. Gly-βMCA was mixed with a chow diet (0.1% w/w) to achieve ∼160 mg/kg/day dose, which was calculated based on a 4 g/day food intake by a 25 g mouse. AAV-FGF15 (catalog no.: AA08-Mm2060, under hepatocyte-specific albumin promoter) was purchased from GeneCopoeia (Rockville, MD). A dose of 4 × 10^10^ GC/mouse was given via tail vein injection. All treatments were initiated when mice were 8 weeks old and lasted for 4 weeks. Tissues and blood samples were collected from mice following a 6-h fast from 9 AM to 3 PM. WT mice, untreated *Cyp2c70* KO mice, and Gly-βMCA-treated *Cyp2c70* KO mice were part of a recently published study by our group (24). These samples were combined with new samples from the AAV-FGF15 and the Gly-βMCA + AAV-FGF15 group, and all assays were measured in the same experiment using these samples, with the exception of the body weight (BW) and liver weight (LW) data and the bile acid composition analysis by LC-MS, where previously published values of the WT mice, untreated *Cyp2c70* KO mice, and Gly-βMCA-treated *Cyp2c70* KO mice were used with the values of the AAV-FGF15 group and the Gly-βMCA + AAV-FGF15 group for analysis (24). Animals received humane care according to the criteria outlined in the “Guide for the Care and Use of Laboratory Animals.” All animal studies were approved by the Institutional Animal Care and Use Committee of the University of Oklahoma Health Sciences (approval no.: 22-072-EAFHI).

### Bile acid analysis

This analysis was performed as described in detail previously (13). Briefly, bile acids were extracted in 95% ethanol from 50 to 100 mg liver tissue, whole gallbladder bile, whole small intestine with content, and dried feces. Fecal samples from an individual mouse were collected during the last week of the 4 weeks of treatment by placing a mouse in a jar briefly. Total bile acid concentration in the bile acid extracts was measured by a bile acid assay kit. Total liver bile acid amount was calculated based on liver bile acid concentration and total LW. The bile acid pool was calculated as the sum of total bile acids in the whole liver, gallbladder, and small intestine. Bile acid species in various bile acid extracts were measured by an LC-MS method (13). The bile acid pool hydrophobicity index was calculated based on a previous report (26). The hydrophobicity index of αMCA, βMCA, and LCA was not available, and the hydrophobicity index values of their taurine conjugates were used in the calculation.

### Real-time PCR

Total liver RNA was purified with Trizol reagent (Sigma-Aldrich, St Louis, MO). Reverse transcription was performed by using the Oligo dT primer and SuperScript III reverse transcriptase (ThermoFisher Scientific, Grand Island, NY). Real-time PCR was performed on a Bio-Rad CFX384 Real-time PCR system with iQ SYBR Green Supermix (Bio-Rad, Hercules, CA). The comparative CT (Ct) method was used to determine the relative mRNA expression with GAPDH used for normalization. The control group was arbitrarily set as “1.”

### Liver pathology

Liver fibrosis was evaluated by Sirius red stain of liver sections. Ductular reaction was evaluated by CK19 immunohistochemistry stain of liver sections. The positive staining area of 4x images was quantified by using Fiji ImageJ software (the National Institutes of Health). Liver ductular reaction/proliferation was also evaluated by a clinical liver pathologist in a blinded fashion. The scoring of bile ductular reaction/proliferation was adapted as previously described (27). Score 0 if there are less than five bile ducts per portal tract; score 1 if there are five to nine bile ducts per portal tract; score 2 if there are equal to or greater than 10 bile ducts per portal tract; and score 3 if there are equal to or greater than 10 bile ducts per portal tract with prominent lobular extension.

### Microbiome analysis

Fecal DNA was extracted using a QIAamp PowerFecal Pro DNA kit (catalog no.: 51804) from Qiagen (Germantown, MD). The 16S sequencing (amplicon region: V3-V4) was performed on an Illumina NovaSeq 6000 platform by Novogene Corporation, Inc (Sacramento, CA). Sequencing libraries were generated, and indexes were added. The library was checked with Qubit and real-time PCR for quantification and bioanalyzer for size distribution detection. Quantified libraries were pooled and sequenced. For bioinformatics analysis, paired-end reads were assigned to samples based on their unique barcodes and truncated by cutting off the barcodes and primer sequences. The whole process was performed through Python (version 3.6.13), and adaptors were removed through cutadapt (version 3.3). Paired-end reads were merged using FLASH (version 1.2.11, http://ccb.jhu.edu/software/FLASH/). Quality filtering on the raw tags was performed using the fastp (version 0.23.1) software to obtain high-quality clean tags. The tags were compared with the reference database (Silva database [16S/18S], https://www.arb-silva.de/;UniteDatabase [ITS], https://unite.ut.ee/) to detect chimera sequences. And the effective tags were obtained by removing the chimera sequences with the vsearch package (version 2.16.0, https://github.com/torognes/vsearch). Sequence analyses were performed by Uparse software (Uparse v7.0. 1001, http://drive5.com/uparse/). Operational taxonomic unit abundance information was normalized using a standard of sequence number corresponding to the sample with the least sequences. Subsequent analysis of α diversity and β diversity was performed based on this output normalized data. The α diversity was evaluated by the Shannon index, which was calculated with QIIME (version 1.9.1) and displayed with R software (version 4.0.3). Rank abundance curve was plotted by using the RColorBrewer package in R. The β diversity on weighted unifrac was calculated by QIIME software (version 1.9.1). Principal coordinate analysis was displayed by ade4 package and ggplot2 package in R software (version 4.0.3). The dataset has been deposited in the National Center for Biotechnology Information GenBank (BioProject accession: PRJNA1208542).

### Statistics

All results were expressed as mean ± SEM. GraphPad Prism 10 was used for statistical analysis. A *P* < 0.05 was considered statistically significant: “∗”<0.05; “∗∗”<0.01; “∗∗∗”<0.001; and “∗∗∗∗”<0.0001.

## Results

### Combining Gly-βMCA with AAV-FGF15 is highly effective in reducing bile acid pool

**Fig. 1:**
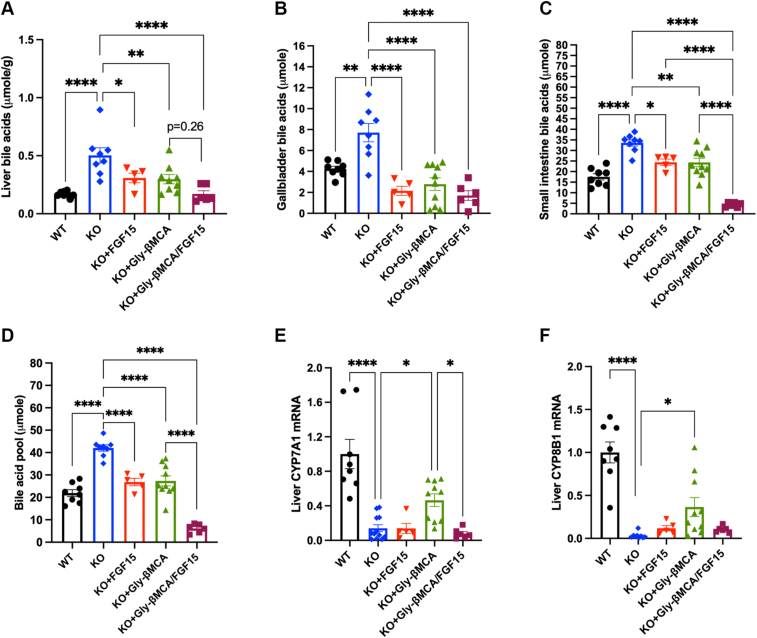
Gly-βMCA and AAV-FGF15 combination effectively reduced bile acid pool in female *Cyp2c70* KO mice. Female *Cyp2c70* KO mice at 8 weeks of age were treated with Gly-βMCA and/or AAV-FGF15 as indicated for 4 weeks. Female 12-week-old WT mice were included in the analysis. A–D: Tissue bile acid content and total bile acid pool. n = 5–10. E and F: Liver mRNA expression. n = 5–12. All results are expressed as mean ± SEM. One-way ANOVA and Tukey post hoc test were used for all statistical analyses. A *P* < 0.05 was considered statistically significant. “∗”<0.05; “∗∗”<0.01; “∗∗∗”<0.001; and “∗∗∗∗”<0.0001.

To test if adding Gly-βMCA to AAV-FGF15 can achieve additional reduction in bile acid pool and hepatic bile acid burden, we administered AAV-FGF15 to 8-week-old female *Cyp2c70* KO mice, which was an effective approach we and others used to mimic FGF19 analog treatment in mice (13, 28, 29) These *Cyp2c70* KO mice were subsequently treated with 160 mg/kg/day Gly-βMCA for 4 weeks, and the effects on bile acid metabolism were compared with untreated controls, and *Cyp2c70* KO mice were treated with either single agent. We found that *Cyp2c70* KO mice had significantly elevated hepatic bile acid accumulation and a larger total bile acid pool than WT mice (Fig. 1A–D). Interestingly, while treating *Cyp2c70* KO mice with either AAV-FGF15 or Gly-βMCA alone reduced the total bile acid pool by ∼35%, combining the two agents reduced the bile acid pool by more than 80% (Fig. 1D). Liver FGF15 mRNA expression was readily detectable by real-time PCR with an average Ct value of ∼20 in AAV-FGF15-treated mice but was not detected in other groups of mice because of lack of endogenous FGF15 expression in mouse hepatocytes (Supplemental Fig. 1A) (30). Hepatic CYP7A1 mRNA was significantly lower than in WT mice (Fig. 1E), which was consistent with higher hepatic bile acid concentration. Gly-βMCA treatment significantly increased hepatic CYP7A1 mRNA, presumably because of reduced hepatic bile acid concentration and FXR antagonism. While AAV-FGF15 did not further reduce CYP7A1 mRNA, it fully blocked CYP7A1 mRNA induction when combined with Gly-βMCA treatment in *Cyp2c70* KO (Fig. 1E). Hepatic sterol 12α-hydroxylase (CYP8B1) mRNA was also markedly lower in *Cyp2c70* KO mice than in WT mice, and AAV-FGF15 in the combination treatment prevented Gly-βMCA-mediated induction of CYP8B1 mRNA in *Cyp2c70* KO mice (Fig. 1F). Sterol 27-hydroxylase (CYP27A1), oxysterol 7α-hydroxylase (CYP7B1), and liver receptor homolog-1 mRNA expressions were not significantly different among all groups (Supplemental Fig. 1B–D). Measurement of the mRNA expression of other bile acid-sensitive genes revealed that liver small heterodimer partner and organic solute transporter β, which are known to be induced by bile acids and FXR (31, 32) were higher in *Cyp2c70* KO mice than WT mice, and their mRNA was decreased by all three treatments (Supplemental Fig. 1E–F). In contrast, the mRNA of sodium-taurocholate cotransporting polypeptide, which is repressed by bile acid and FXR (33) was lower in *Cyp2c70* KO mice than WT mice, and its expression was restored upon the combination treatment (Supplemental Fig. 1G). The mRNA of the bile salt export pump was significantly lower in the combination treatment group compared with untreated *Cyp2c70* KO mice (Supplemental Fig. 1H), possibly because of significantly lower bile acid levels in these mice (34). The mRNA of multidrug resistance-associated protein 3, which mediates basolateral bile acid efflux, was induced in the FGF15 group by an unknown mechanism (Supplemental Fig. 1I). Ileal FGF15 mRNA was reduced in all three treatment groups (Supplemental Fig. 1J). These gene expression changes were generally consistent with the bile acid pool changes and the FXR antagonism effect of Gly-βMCA. In summary, these results demonstrated that the AAV-FGF15 and Gly-βMCA combination was highly effective in reducing the total bile acid pool in *Cyp2c70* KO mice.

### The Gly-βMCA and AAV-FGF15 combination treatment was more effective than single treatment in reducing bile acid hydrophobicity

**Fig. 2:**
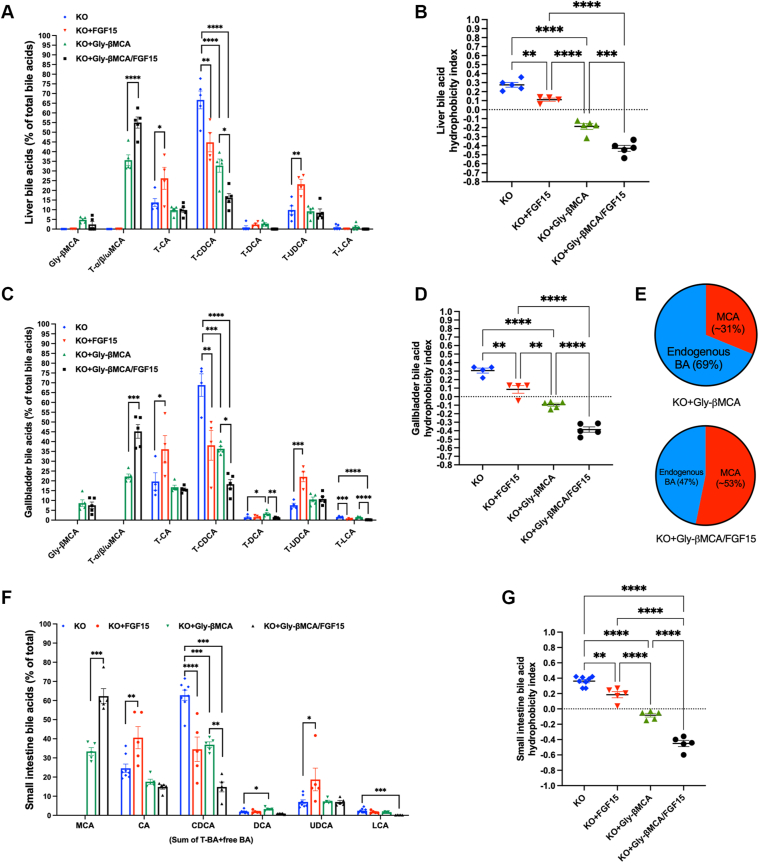
Gly-βMCA and AAV-FGF15 combination effectively reduced enterohepatic bile acid hydrophobicity in female *Cyp2c70* KO mice. Female *Cyp2c70* KO mice at 8 weeks of age were treated with Gly-βMCA and/or AAV-FGF15 as indicated for 4 weeks. Bile acid species were measured by the LC-MS method. A: Bile acid composition of liver bile acid extracts. B: Liver bile acid hydrophobicity index. C: Gallbladder bile acid composition. D: Gallbladder bile acid hydrophobicity index. E: Total MCA abundance (% of the sum of Gly-βMCA, T-⍺MCA, T-βMCA, and T-ωMCA in the sum of all measured bile acids) in the gallbladder bile of female Cyp2c70 KO mice treated with Gly-βMCA or the combination treatment as shown in “C.” F: Small intestine bile acid composition. The relative abundance of each bile acid is the sum of taurine-conjugated (T-BA) and unconjugated bile acid (free BA). G: Small intestine bile acid hydrophobicity index. All results are expressed as mean ± SEM. n = 4–8. One-way ANOVA and Tukey post hoc test were used for all statistical analyses. A *P* < 0.05 was considered statistically significant. “∗”<0.05; “∗∗”<0.01; “∗∗∗”<0.001; and “∗∗∗∗”<0.0001.

We next analyzed the treatment effects on bile acid composition in liver, gallbladder bile, and small intestine bile acid extracts by using LC-MS methods. The bile acid compositions in liver tissues and gallbladder bile were highly similar among all groups (Fig 2A, C). AAV-FGF15 treatment increased the relative abundance of T-CA and T-UDCA and decreased the relative abundance of T-CDCA (Fig 2A, C). These changes of bile acid composition resulted in a slightly decreased bile acid hydrophobicity than untreated *Cyp2c70* KO controls (Fig 2B, D). Gly-βMCA treatment caused enrichment of Gly-βMCA and T-MCA species and decreased the relative abundance of T-CDCA, resulting in significantly reduced bile acid hydrophobicity (Fig. 2A–D). In comparison, the combination treatment caused a higher enrichment of T-MCA and a further reduction of the relative abundance of T-CDCA, resulting in a significantly lower bile acid hydrophobicity than either single treatment (Fig. 2A–E). The further reduction of T-CDCA by the combination treatment was likely caused by reduced hepatic CYP7A1 expression by FGF15 that limited endogenous bile acid synthesis and Gly-βMCA-mediated inhibition of gut endogenous T-CDCA absorption, which subsequently increased the relative abundance of T-MCA. The small intestine bile acid composition was highly similar to that of the liver and gallbladder bile acid composition of the same group (Fig. 2F), and the combination treatment resulted in the lowest bile acid hydrophobicity among all three treatment groups (Fig. 2G). These results demonstrated that the AAV-FGF15 and Gly-βMCA combination not only decreased total bile acid pool but also was effective in reducing bile acid pool hydrophobicity, which was not achieved by the AAV-FGF15 and ASBT inhibitor combination (13).

### The Gly-βMCA and AAV-FGF15 combination improved liver injury and portal fibrosis in female *Cyp2c70* KO mice

**Fig. 3:**
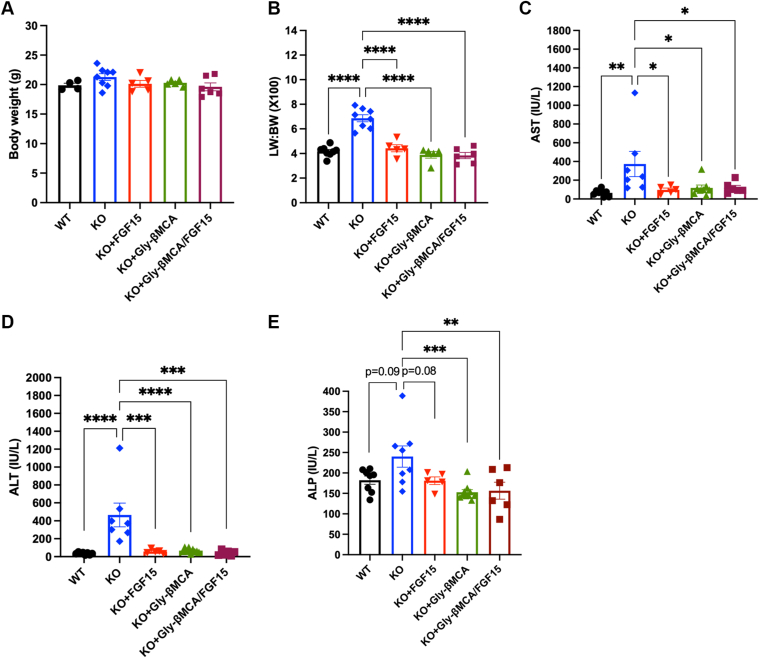
Single and combination treatments reduced serum liver injury markers in female *Cyp2c70* KO mice. Female *Cyp2c70* KO mice at 8 weeks of age were treated with Gly-βMCA and/or AAV-FGF15 as indicated for 4 weeks. Female 12-week-old WT mice were included in the analysis. A: BW. B: LW to BW ratio. C and D: Serum aspartate aminotransferase and alanine aminotransferase concentration. E: Serum alkaline phosphatase concentration. n = 5–9. All results are expressed as mean ± SEM. One-way ANOVA and Tukey post hoc test were used for all statistical analyses. A *P* < 0.05 was considered statistically significant. “∗”<0.05; “∗∗”<0.01; “∗∗∗”<0.001; and “∗∗∗∗”<0.0001.

**Fig. 4:**
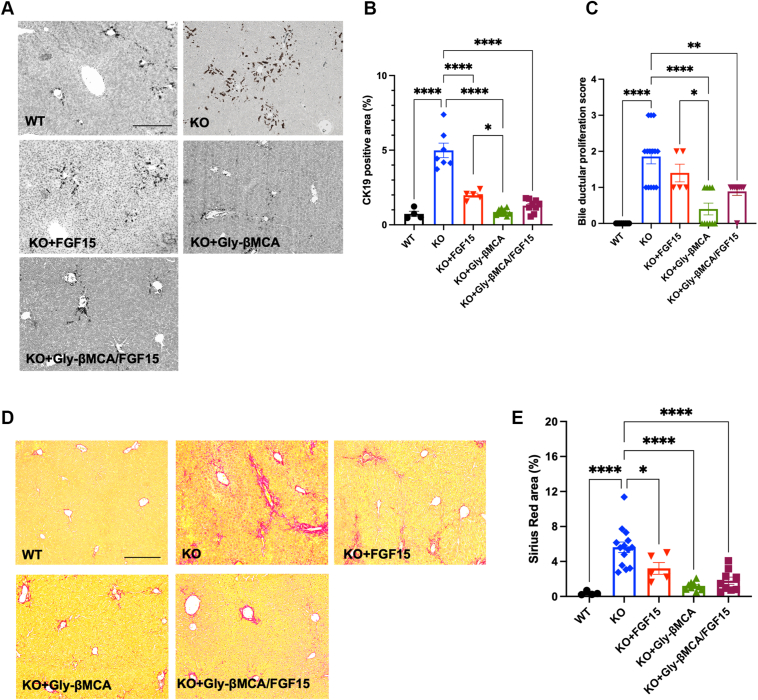
Single and combination treatments improved ductular reaction and portal fibrosis in female *Cyp2c70* KO mice. Female *Cyp2c70* KO mice at 8 weeks of age were treated with Gly-βMCA and/or AAV-FGF15 as indicated for 4 weeks. Female 12-week-old WT mice were included in the analysis. A: Representative images of CK-19 immunohistochemistry. The scale bar represents 250 μm. B: Quantification of CK-19 positive area. n = 4–10. C: Bile duct proliferation score. n = 5–14. D: Representative images of liver Sirius red stain. The scale bar represents 250 μm. E: Quantification of Sirius red-positive area. n = 4–14. All results are expressed as mean ± SEM. One-way ANOVA and Tukey post hoc test were used for all statistical analyses. A *P* < 0.05 was considered statistically significant. “∗”<0.05; “∗∗”<0.001; and “∗∗∗∗”<0.0001.

We next investigated the relative effectiveness of each treatment in attenuating hepatobiliary injury and fibrosis in the *Cyp2c70* KO mice. The BW was not significantly different among all groups, whereas the LW:BW ratio was significantly higher in *Cyp2c70* KO mice than in WT mice (Fig. 3A–B). All three treatments fully normalized the LW:BW ratio in *Cyp2c70* KO mice with that of WT mice (Fig. 3B). Similarly, all three treatments fully reduced the serum aspartate aminotransferase and alanine aminotransferase in *Cyp2c70* KO mice to levels comparable to WT mice (Fig. 3C–D). Serum alkaline phosphatase, a marker of biliary injury, was more elevated in *Cyp2c70* KO mice than in WT mice and was lowered by all treatments to a similar level in *Cyp2c70* KO mice (Fig. 3E). While the serum hepatobiliary injury markers were similarly reduced by all three treatments in *Cyp2c70* KO mice, Gly-βMCA treatment was significantly more effective than AAV-FGF15 treatment in reducing bile ductular reaction in *Cyp2c70* KO mice, as evidenced by significantly lower CK19-positive area and bile ductular proliferation score in Gly-βMCA-treated mice than untreated controls and AAV-FGF15-treated mice (Fig. 4A–C). The combination treatment did not cause a further reduction of ductular reaction compared with either single treatment. Furthermore, Gly-βMCA treatment also showed a tendency to be more effective in reducing portal fibrosis than AAV-FGF15 and the combination treatment in *Cyp2c70* KO mice, although no statistically significant difference was noted among the three treatment groups (Fig. 4D–E). These data suggest that adding FGF15 to Gly-βMCA did not provide additional therapeutic improvement over Gly-βMCA single treatment in *Cyp2c70* KO mice. This is possibly because Gly-βMCA alone was highly effective in reducing ductular reaction and fibrosis in *Cyp2c70* KO mice, leaving little room for further improvement by the combination treatment despite a much smaller and more hydrophilic bile acid pool.

### The Gly-βMCA and AAV-FGF15 combination remodeled fecal bile acid composition and effectively decreased fecal bile acid hydrophobicity

**Fig. 5:**
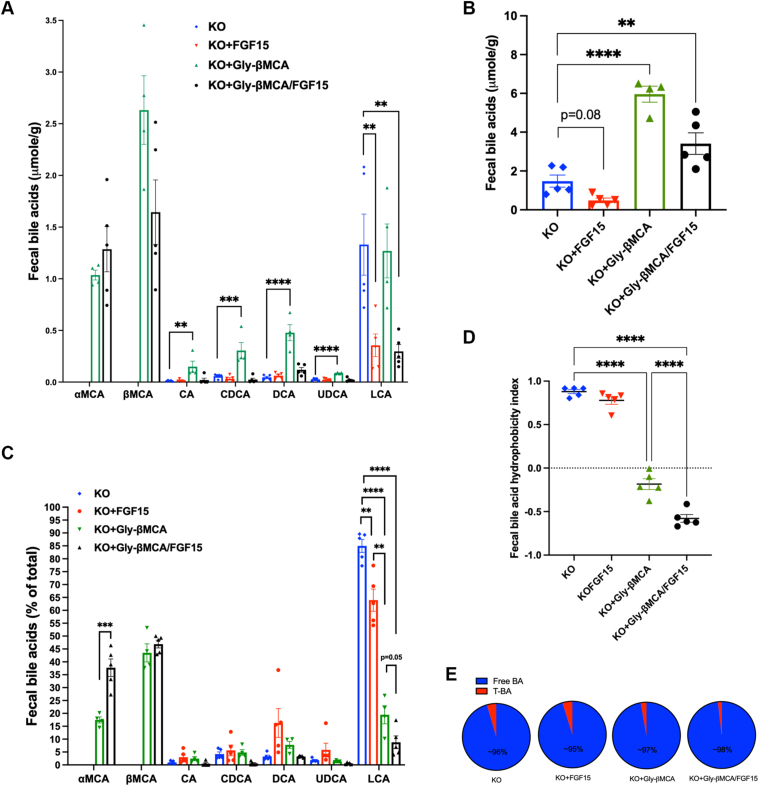
Gly-βMCA and AAV-FGF15 combination effectively reduced fecal bile acid hydrophobicity in female *Cyp2c70* KO mice. Female *Cyp2c70* KO mice at 8 weeks of age were treated with Gly-βMCA and/or AAV-FGF15 as indicated for 4 weeks. A: Fecal bile acid species normalized to fecal sample weight. B: Total fecal bile acid amount normalized to fecal sample weight. C: Fecal bile acid composition. D: Fecal bile acid hydrophobicity index. E: Relative abundance of fecal unconjugated bile acids (free BA) and taurine-conjugated bile acids (T-BA). All results are expressed as mean ± SEM. n = 4–5. One-way ANOVA and Tukey post hoc test were used for all statistical analyses. A *P* < 0.05 was considered statistically significant. “∗”<0.05; “∗∗”<0.01; “∗∗∗”<0.001; and “∗∗∗∗”<0.0001.

Our previous study revealed that Gly-βMCA inhibited the gut absorption of endogenous bile acids and promoted their fecal excretion in *Cyp2c70* KO mice, which was an important mechanism by which Gly-βMCA administration paradoxically reduced the bile acid pool instead of expanding it (23, 24). In this study, we further investigated how combining AAV-FGF15 and Gly-βMCA modulated fecal bile acid composition, which closely reflected large intestine bile acid exposure. The highly hydrophobic and toxic bile acid LCA was the predominant fecal bile acid species in the *Cyp2c70* KO mice (Fig. 5A), which was largely attributed to T-CDCA, the precursor of LCA, being the predominant bile acid in the gallbladder bile and small intestine (Fig 2A, D), and that the fact that the secondary bile acid DCA, which was derived from CA, was more efficiently absorbed, whereas LCA was mostly excreted into feces. While AAV-FGF15 treatment decreased bile acid pool (Fig. 1D) and fecal bile acid excretion (Fig. 5B), Gly-βMCA treatment promoted the excretion of not only MCA but also endogenous bile acids CA, CDCA, DCA, and UDCA and increased total fecal bile acids by ∼4-fold (Fig. 5A–B). Interestingly, the combination treatment also increased fecal bile acid loss by more than 2-fold (Fig. 5B). As such, the fecal bile acid excretion was maintained at a higher level in mice treated with the combination despite a markedly smaller endogenous bile acid pool size (Fig. 1D). Furthermore, αMCA and βMCA together accounted for more than 80% of fecal bile acids, and LCA was reduced to less than 10% of total fecal bile acids by the combination treatment (Fig. 5C), which may also be partly attributed to significantly lower small intestine CDCA, the precursor of LCA (Fig. 2D). As a result of these treatment-dependent changes of fecal bile acid composition, we found that AAV-FGF15 treatment slightly decreased the relative abundance of LCA but did not significantly decrease fecal bile acid hydrophobicity (Fig. 5C-D). Gly-βMCA treatment increased the relative abundance of fecal MCA and decreased the relative abundance of fecal LCA, resulting in a significantly lower fecal bile acid hydrophobicity (Fig. 5C–D). In comparison, the combination treatment was more effective in enriching fecal MCA and decreasing the relative abundance of LCA, resulting in an even lower bile acid hydrophobicity index than the Gly-βMCA treatment group (Fig. 5C–D). Last, we found that the fecal bile acids were predominantly in unconjugated form in all groups (Fig. 5E), suggesting that bile acid deconjugation was carried out largely to completion despite significantly increased transintestine bile acid flux in mice treated with Gly-βMCA and the combination as reflected by higher fecal bile acid excretion (Fig. 5B). Taken together, these results suggested that the combination treatment sustained bile acid excretion despite a smaller bile acid pool and was more effective than AAV-FGF15 and Gly-βMCA single treatment in reducing colon exposure to hydrophobic bile acids.

### Gly-βMCA and AAV-FGF15 differentially regulated the microbiome in female *Cyp2c70* KO mice

**Fig. 6:**
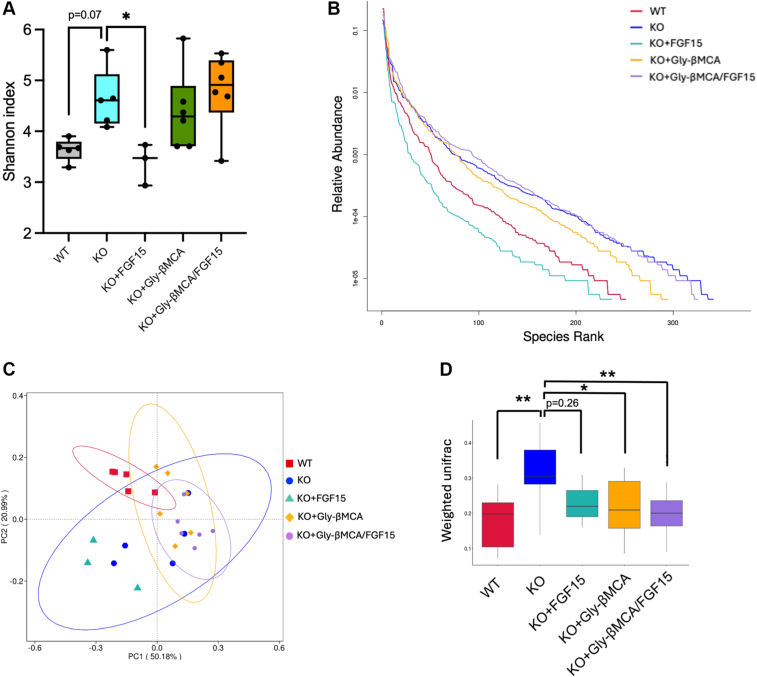
Gly-βMCA and AAV-FGF15 combination remodels microbiome in female *Cyp2c70* KO mice. Female *Cyp2c70* KO mice at 8 weeks of age were treated with Gly-βMCA and/or AAV-FGF15 as indicated for 4 weeks. Female 12-week-old WT mice were included in the analysis. A: α diversity Shannon index. Statistical analysis was performed with one-way ANOVA and Dunnett post hoc test. B: Rank abundance curve. *Y*-axis: total abundance is set as ”1.” C. Principal coordinates analysis plot. D: β diversity weighted unifrac plot. Statistical analysis was performed with Kruskal-Wallis test. n = 3–6. A *P* < 0.05 was considered statistically significant. “∗”<0.05; “∗∗”<0.01; “∗∗∗”<0.001; and “∗∗∗∗”<0.0001.

To obtain further insights into the interaction of bile acid metabolism with gut microbiome, we next performed 16S sequencing of fecal microbiome. We found that *Cyp2c70* KO mice showed increased microbiome α diversity than WT mice as indicated by the Shannon index (Fig. 6A), which was attributed to both the increased number of bacterial species and evenness (Fig. 6B). AAV-FGF15 treatment significantly decreased microbiome α diversity, whereas the Gly-βMCA and the combination treatment did not affect microbiome α diversity in *Cyp2c70* KO mice (Fig. 6A–B). Therefore, decreased colon bile acid exposure caused by AAV-FGF15 treatment correlated with a decrease of microbiome α diversity. Furthermore, Gly-βMCA and the combination treatment, which increased colon exposure to hydrophilic bile acids, did not affect microbiome α diversity in *Cyp2c70* KO mice.

**Fig. 7:**
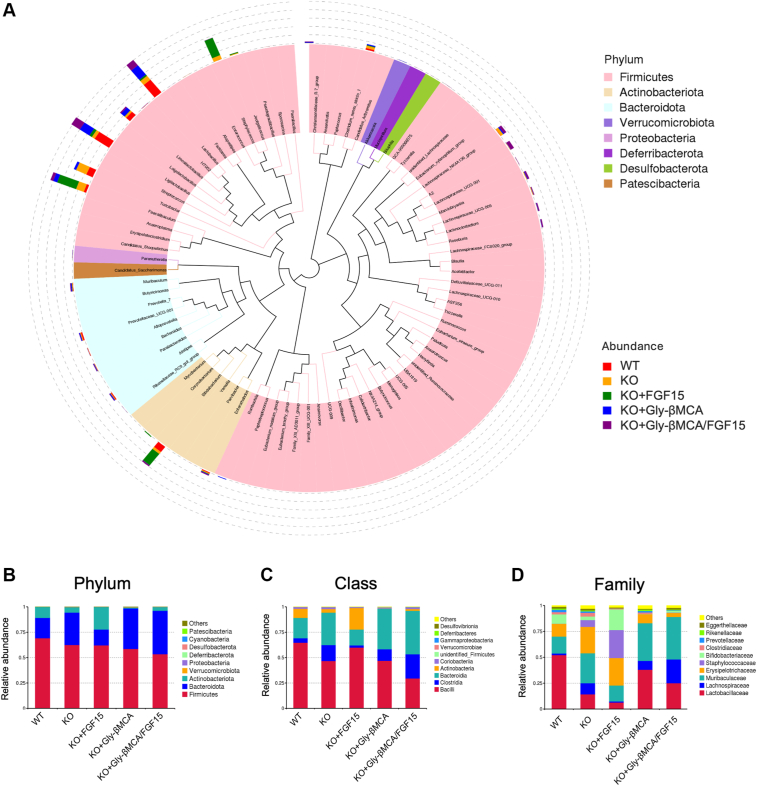
Taxonomic distribution of microbiome in WT and *Cyp2c70* KO mice. Female *Cyp2c70* KO mice at 8 weeks of age were treated with Gly-βMCA and/or AAV-FGF15 as indicated for 4 weeks. Female 12-week-old WT mice were included in the analysis. 16S sequencing was performed to analyze fecal microbiome composition. n = 3–6. A: Circular phylogenetic tree plot. Distribution at the genus level. B–D: Barplot illustration of the relative mean abundance at the rank of phylum “B,” class “C,” and family “D.”

**Fig. 8:**
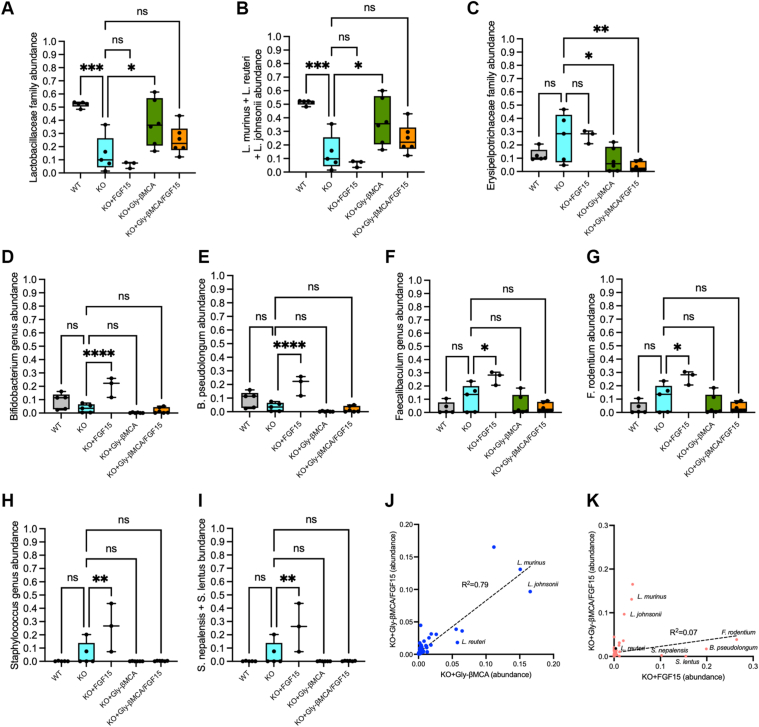
Gly-βMCA and AAV-FGF15 treatments enriched distinct populations of beneficial microbiota in female *Cyp2c70* KO mice. Female *Cyp2c70* KO mice at 8 weeks of age were treated with Gly-βMCA and/or AAV-FGF15 as indicated for 4 weeks. Female 12-week-old WT mice were included in the analysis. 16S sequencing was performed to analyze fecal microbiome composition. n = 3–6. A–I: Relative abundance of microbiota at the ranks of family, genus, or species. “B” shows the sum of the relative abundance of *Lactobacillus murinus*, *Lactobacillus johnsonii*, and *Lactobacillus reuteri*. “I” shows the sum of the relative abundance of the *Staphylococcus lentus* and *Staphylococcus nepalensis* species. Results are expressed as mean ± SEM. One-way ANOVA and Dunnett post hoc test were used for statistical analysis. A *P* < 0.05 was considered statistically significant. “∗”<0.05; “∗∗”<0.01; “∗∗∗”<0.001; and “∗∗∗∗”<0.0001. ns, not significant. J–K: Correlation of the top 100 abundant species in the combination treatment group versus either the Gly-βMCA group or the AAV-FGF15 group. R: Pearson correlation coefficient. In all plots, the total abundance is set as “1.”

Analysis of β diversity revealed that *Cyp2c70* KO mice had a clearly distinct microbial composition than the WT mice (Fig. 6C–D). Furthermore, mice treated with Gly-βMCA and the combination treatment, but not the AAV-FGF15, had significantly altered microbiome β diversity than untreated *Cyp2c70* KO mice (Fig. 6C–D). These data implied that bile acid composition and hydrophobicity may have a significant impact on gut microbiome composition. To obtain further insights, we analyzed the microbial composition by focusing on the top abundant groups within each taxonomic rank, aiming to detect how major gut microbiome taxonomic groups were altered in *Cyp2c70* KO mice and the subsequent treatment-dependent effects in *Cyp2c70* KO mice. As expected, Firmicutes, Bacteroidota, and Actinobacteria are the three predominant gut bacterial phyla in both WT and *Cyp2c70* KO mice, and their relative abundance was not significantly different between WT and *Cyp2c70* KO mice (Fig. 7A–B). However, of the class Bacilli within the Firmicutes phylum, we found that *Cyp2c70* KO mice showed markedly reduced abundance of the Lactobacillaceae family compared with WT mice, and Gly-βMCA treatment significantly increased the Lactobacillaceae family in *Cyp2c70* KO mice (Fig. 7B–D, Fig. 8A). Notably, further analysis showed that the contributors of the Lactobacillaceae family changes were narrowed down to three predominant bacteria species: *Lactobacillus murinus* of the Ligilactobacillus genus and *Lactobacillus johnsonii* and *Lactobacillus reuteri* of the *Lactobacillus* genus (Fig. 8A–B). These were considered beneficial bacteria and commonly used probiotics that had been shown to promote the host's gut health and alleviate chronic liver diseases (35, 36, 37, 38). On the contrary, Gly-βMCA significantly decreased the relative abundance of the Erysipelotrichaceae family of the Firmicutes phylum in *Cyp2c70* KO mice (Fig. 8C). The relative abundance of the Erysipelotrichaceae family was ∼2-fold higher in *Cyp2c70* KO mice than the WT mice although the increase was not statistically significant (*P* = 0.14) (Fig. 8C). The functions of the Erysipelotrichaceae family microbiota were less well understood, but its abundance has previously been positively associated with intestine inflammation and chronic liver diseases in human patients (39). Gly-βMCA did not significantly alter the rest of the top 10 abundant families within the Firmicutes, the Bacteroidota, or the Actinobacteria phyla, suggesting a selective effect of Gly-βMCA treatment toward the Lactobacillaceae and Erysipelotrichaceae family bacteria.

In comparison, AAV-FGF15 treatment, which decreased fecal bile acid amount but not hydrophobicity, did not significantly alter the relative abundance of the Lactobacillaceae and Erysipelotrichaceae families that were sensitive to Gly-βMCA treatment (Fig 8A, C). However, we found that the most prominent changes caused by AAV-FGF15 were increased relative abundance of the *Bifidobacterium* genus of the Bifidobacteriaceae family within the Actinobacteria phylum, the *Faecalibaculum* genus of the Erysipelotrichaceae family within the Firmicutes phylum, and the *Staphylococcus* genus of the *Staphylococcaceae* family within the *Firmicutes* phylum, which was largely attributed to changes of the *Bifidobacterium pseudolongum* species (Fig. 8D–E), the *Faecalibaculum rodentium* species (Fig. 8F–G), and the sum of the *Staphylococcus lentus* and *Staphylococcus nepalensis* species (Fig. 8H–I), respectively. Among them, *Bifidobacterium* and *Faecalibaculum* bacteria species have been shown to act as probiotics to provide beneficial effects against liver diseases (40). Limited studies suggested that some species in the *Staphylococcus genus* have been recognized as important pathogens that cause infection in chronic liver diseases (41), but the enrichment of *S*. *lentus* and *S*. *nepalensis* did not positively correlate with liver injury severity in AAV-FGF15-treated *Cyp2c70* KO mice. The abundance of these bacteria was not significantly altered by the Gly-βMCA treatment in *Cyp2c70* KO mice (Fig. 8D–I). In general, the effects of the Gly-βMCA and AAV-FGF15 combination on microbial composition more closely resembled the effects of the Gly-βMCA treatment but not the effects of the AAV-FGF15 treatment in *Cyp2c70* KO mice (Fig. 6C). In further support, the top 100 most abundant species in the combination treatment group strongly correlated with that of the Gly-βMCA treatment group (*R*^2^ = 0.79) (Fig. 8J) but poorly correlated with the AAV-FGF15 group (*R*^2^ = 0.07) (Fig. 8K), although the increase of the Lactobacillaceae family bacteria caused by the combination treatment did not reach statistical significance compared with untreated *Cyp2c70* KO mice (Fig. 8A–B). The similar effects of the Gly-βMCA and AAV-FGF15 combination on microbial composition were in line with the similar effects of the Gly-βMCA treatment and the combination treatment on fecal bile acid excretion (Fig. 5B), composition (Fig. 5C), and hydrophobicity (Fig. 5D). In summary, treatment-dependent changes in bile acid composition and hydrophobicity were closely linked to gut microbiome remodeling, with beneficial lactic acid bacteria of the Lactobacillaceae family negatively correlated with liver injury severity.

## Discussion

This study is our continued effort to develop an effective therapeutic strategy that simultaneously targets hepatic bile acid synthesis and intestine bile acid absorption to alleviate bile acid toxicity in cholestasis. Based on this concept, our previous study has demonstrated that combining AAV-FGF15 with an ASBT inhibitor was a highly effective approach to achieve a significantly higher degree of bile acid pool size reduction than either single treatment in *Cyp2c70* KO mice (13). Further testing of the Gly-βMCA and AAV-FGF15 combination was based on our recent finding that Gly-βMCA exhibited several unique anticholestasis properties (24). Specifically, the poorly absorbed Gly-βMCA acted as an inhibitor of endogenous bile acid absorption in *Cyp2c70* KO mice, which provided the molecular basis for replacing the ASBT inhibitor with Gly-βMCA in this combination. In addition, the majority of the administered Gly-βMCA followed a gut microbiome deconjugation-dependent absorption route to enter the endogenous bile acid pool, and T-MCAs derived from Gly-βMCA not only decreased bile acid pool hydrophobicity but also acted as FXR antagonists to induce hepatic CYP7A1, which was an undesirable effect in cholestasis treatment (24). By adding AAV-FGF15, we were able to fully prevent CYP7A1 induction by Gly-βMCA, which was likely a major mechanism underlying the markedly reduced bile acid pool by this combination therapy than either single agent. In comparison to the effect of the AAV-FGF15 and ASBT inhibitor combination, we found that the Gly-βMCA and AAV-FGF15 combination also caused the bile acid pool to be highly enriched with T-MCA, resulting in a significantly lower bile acid hydrophobicity. It was noted that fecal bile acids were also highly enriched with MCA species upon the Gly-βMCA and AAV-FGF15 combination treatment, which indicated that colon exposure to hydrophobic bile acids was significantly reduced in the treated mice. These effects were not achieved by the AAV-FGF15 and ASBT inhibitor combination in *Cyp2c70* KO mice (13), suggesting that the Gly-βMCA and AAV-FGF15 combination may have several advantages over the AAV-FGF15 with ASBT inhibitor combination.

The close interaction of bile acid metabolism and gut microbiome is well documented. A recent study revealed that germ-free *Cyp2c70* KO mice showed worsened liver disease and reduced neonatal survival rate, which was reversed by subsequent colonization with either mouse or human microbiota (42). As expected, germ-free *Cyp2c70* KO mice did not produce secondary bile acids, including the hydrophilic UDCA, and showed an even higher bile acid hydrophobicity index than conventionalized *Cyp2c70* KO mice (42). These findings showed that the gut microbiome as a whole played a beneficial role for limiting liver bile acid toxicity and ensuring neonatal survival against progressive liver disease in *Cyp2c70* KO mice. By profiling the gut microbiome, we identified treatment-dependent microbiome changes in *Cyp2c70* KO mice. The most notable change was a strong decrease of the highly abundant Lactobacillus family microbiota in *Cyp2c70* KO mice compared with WT mice, which was significantly reversed upon Gly-βMCA treatment. These changes were largely attributed to the combined changes of three predominant species: *L*. *murinus*, *L*. *johnsonii*, and *L*. *reuteri*. These are common probiotics used to promote the growth of beneficial bacteria and provide beneficial effects to the health of the host (40). Enrichment of these bacteria species via therapeutic interventions has been demonstrated by many studies to protect against cholestasis liver injury and other types of chronic liver diseases in experimental models via modulating bile acid metabolism, gut immunity, gut barrier integrity, etc (35,36,37,38) Another notable effect of the Gly-βMCA treatment was a significant decrease of the relative abundance of the Erysipelotrichaceae family, which was reported to be positively associated with intestine inflammation in humans and also elevated in patients with liver diseases (39). In contrast, AAV-FGF15 treatment increased Bifidobacterium and Faecalibaculum bacteria, which have been shown to provide beneficial effects against liver diseases (40). Therefore, AAV-FGF15 and Gly-βMCA modulated distinct groups of gut microbiome, which may possibly be linked to their differential effects on the fecal bile acid excretion, composition, and hydrophobicity. In agreement, the changes of both fecal bile acid abundance and composition and fecal microbiome composition caused by the AAV-FGF15 and Gly-βMCA combination resembled those of the Gly-βMCA treatment but not the AAV-FGF15 treatment. Based on the current literature, bile salt hydrolase activity has been reported in all top 10 most abundant bacterial genera found in WT and *Cyp2c70* KO mice (43). Therefore, it is still unclear how altered microbiome composition may affect the bile salt hydrolase activity in these mice. We found that fecal bile acids were mostly in unconjugated form in all groups, suggesting that none of the treatments limited bile acid deconjugation in the *Cyp2c70* KO mice. We note here that these are correlative data that do not sufficiently establish causal relationships between bile acids, gut microbiome, and liver pathology in the treatment groups. Future longitudinal studies of gut microbiome changes and intervention approaches to manipulate gut microbiome composition are required to delineate how the observed changes in microbiome may in turn impact the treatment outcomes.

Upon evaluating the therapeutic efficacy against hepatobiliary injury and fibrosis, we found that all three treatments reduced hepatomegaly and serum hepatobiliary injury markers to similar levels in the *Cyp2c70* KO mice, and Gly-βMCA treatment appeared to be slightly more effective in preventing bile duct proliferation and portal fibrosis than AAV-FGF15 treatment. However, the Gly-βMCA and AAV-FGF15 combination did not provide further improvement of liver pathology compared with the Gly-βMCA treatment in *Cyp2c70* KO mice. An explanation is that *Cyp2c70* KO mice were highly responsive to the Gly-βMCA monotherapy, leaving little room for further improvement by the combination treatment. Although Gly-βMCA monotherapy only reduced the total bile acid pool by ∼40% as compared with the ∼80% reduction by the Gly-βMCA and AAV-FGF15 combination, the bile acid pool consisted of ∼30% MCA and was hydrophilic, which may explain why Gly-βMCA monotherapy was more effective against hepatobiliary bile acid toxicity than AAV-FGF15, which reduced the total bile acid pool by a similar degree but was less effective in reducing bile acid pool hydrophobicity.

We have recently shown that the abundantly synthesized endogenous MCA species masked the beneficial effect of Gly-βMCA in *Mdr2* KO mice (44), which suggests that study of the therapeutic efficacy of Gly-βMCA as monotherapy or combination therapy in other rodent cholestasis models may have diminished human relevance. In contrast to experimental cholestasis, where a treatment usually elicits a more homogenous response, it is known that human cholestasis varies greatly in response to UDCA, FGF19 analog, or ASBT inhibitor treatment (15, 16, 22). Our study suggests that a combination treatment of FGF19 analog and Gly-βMCA may have clinical implications in conditions where cholestasis patients potentially benefit from enhanced reduction of bile acid pool size and hydrophobicity.

## Data availability

The gut microbiome sequencing data are deposited in GenBank. All other data described in the article are either contained within the article or available upon request.

## Supplemental data

This article contains supplemental data.

## Conflict of interests

The authors declare that they have no conflicts of interest with the contents of this article.
