# Supplementary material for: A Gly-β-muricholic acid and FGF15 combination therapy synergistically reduces “humanized” bile acid pool toxicity in cholestasis mice
Source: J Lipid Res. 2025 Nov 5;66(12):100936. doi: 10.1016/j.jlr.2025.100936 (PMC12702073; doi:10.1016/j.jlr.2025.100936)
Supplement: Supplemental Figure 1 [file mmc1.pdf]

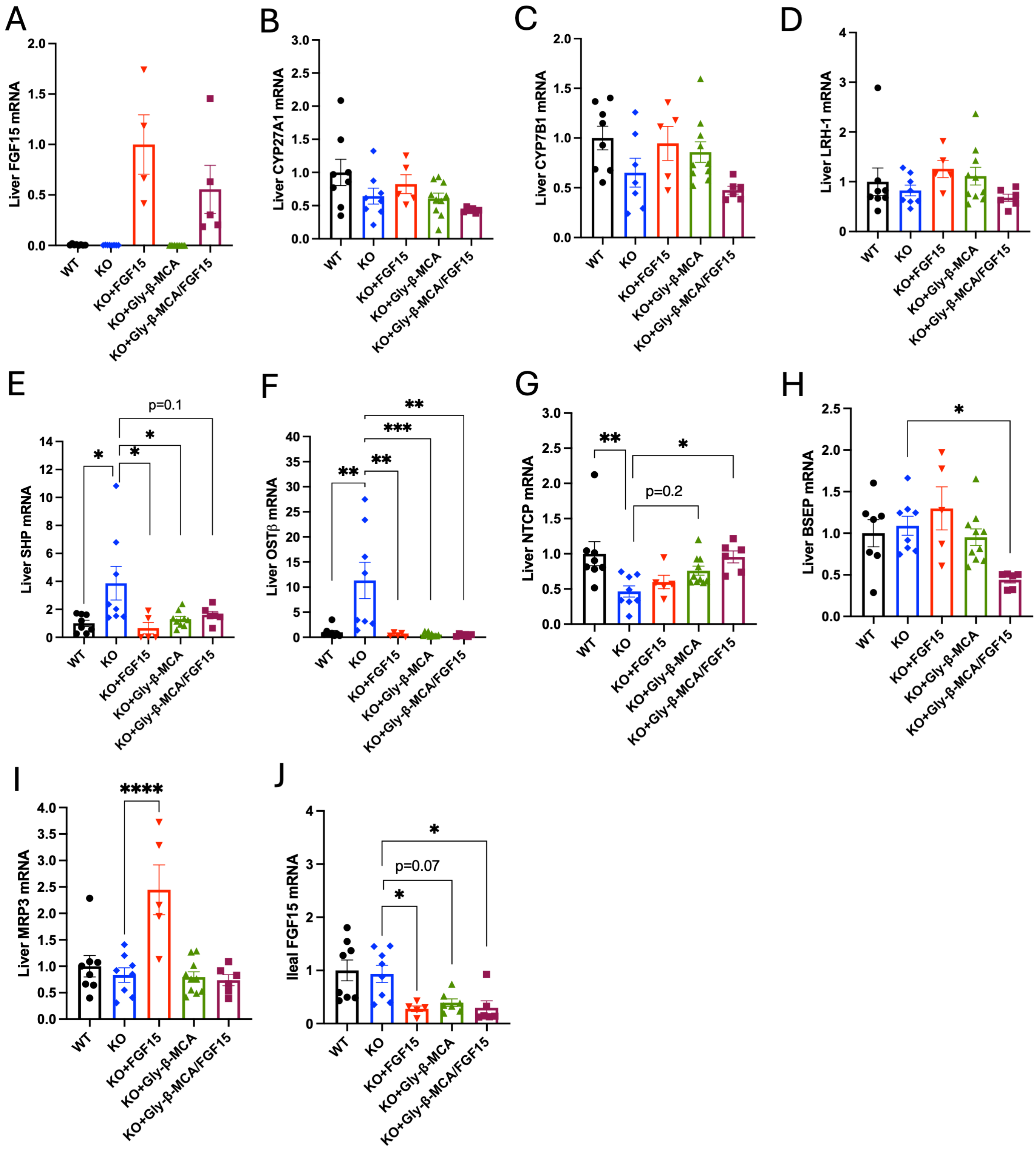

Supplemental Figure 1. Liver and ileal mRNA expression in female *Cyp2c70* KO mice. Female *Cyp2c70* KO mice at 8 weeks of age were treated with Gly-βMCA and/or AAV-FGF15 as indicated for 4 weeks. Female 12 weeks old WT mice were included in the analysis. Real-time PCR was used to measure the relative mRNA expression. **A.** Liver FGF15 mRNA. Because FGF15 is not expressed in mouse hepatocytes, the relative FGF15 expression of KO+FGF15 group was arbitrarily set as "1". **B-I.** Liver mRNA expression. The relative mRNA expression of the WT group was arbitrarily set as "1". **J.** Ileal FGF15 mRNA expression. The relative mRNA expression of the WT group was set as "1". All results are expressed as mean ± SEM. One-way ANOVA and Tukey post hoc test were used for all statistical analysis. A $p < 0.05$ was considered statistically significant. "*", <0.05; "**", <0.01; "***", <0.001; "****" <0.0001.
